# Relationship of post-exercise muscle oxygenation and duration of cycling exercise

**DOI:** 10.1186/s13102-016-0036-y

**Published:** 2016-04-14

**Authors:** Fabian Stöcker, Christoph Von Oldershausen, Florian Kurt Paternoster, Thorsten Schulz, Renate Oberhoffer

**Affiliations:** Center for Teaching and Learning, Technische Universität München, Connollystraße 32, 80809 München, Germany; Department for Biomechanics in Sports, Technische Universität München, München, Germany; Department for Preventive Pediatrics, Technische Universität München, München, Germany

**Keywords:** Muscle oxygenation, Hyperemia, Near-infrared spectroscopy, Interval training, Prior exercise

## Abstract

**Background:**

Aerobic adaptations following interval training are supposed to be mediated by increased local blood supply. However, knowledge is scarce on the detailed relationship between exercise duration and local post-exercise blood supply and oxygen availability. This study aimed to examine the effect of five different exercise durations, ranging from 30 to 240 s, on post-exercise muscle oxygenation and relative changes in hemoglobin concentration.

**Methods:**

Healthy male subjects (*N* = 18) performed an experimental protocol of five exercise bouts (30, 60, 90, 120, and 240 s) at 80 % of peak oxygen uptake $\left({\overset{.}{\mathrm{V}}\mathrm{O}}_{2\mathrm{peak}}\right)$ in a randomized order, separated by 5-min recovery periods. To examine the influence of aerobic fitness, we compared subjects with gas exchange thresholds (GET) above 60 % $\overset{.}{\mathrm{V}}{\mathrm{O}}_{2\mathrm{peak}}$ (GET60+) with subjects reaching GET below 60 % $\overset{.}{\mathrm{V}}{\mathrm{O}}_{2\mathrm{peak}}$ (GET60−). $\overset{.}{\mathrm{V}}{\mathrm{O}}_2$ and relative changes in concentrations of oxygenated hemoglobin, deoxygenated hemoglobin, and total hemoglobin were continuously measured with near-infrared spectroscopy of the vastus lateralis muscle.

**Results:**

Post-exercise oxygen availability and local blood supply increased significantly until the 90-s exercise duration and reached a plateau thereafter. Considering aerobic fitness, the GET60+ group reached maximum post-exercise oxygen availability earlier (60 s) than the GET60− group (90 s).

**Conclusions:**

Our results suggest that (1) 90 s has evolved as the minimum interval duration to enhance local oxygen availability and blood supply following cycling exercise at 80 % $\overset{.}{\mathrm{V}}{\mathrm{O}}_{2\mathrm{peak}}$; whereas (2) 60 s is sufficient to trigger the same effects in subjects with GET60 + .

## Background

High-intensity training (HIT) and high-intensity interval training or aerobic interval training (HIIT or AIT) are commonly accepted stimuli for improving anaerobic [1, 2] and aerobic [3–7] functions. Although the terms HIT and HIIT are sometimes used interchangeably, HIT usually refers to near-maximal or supramaximal exercise intensities (>90 % peak rate of oxygen uptake, $\overset{.}{\mathrm{V}}{\mathrm{O}}_{2\mathrm{peak}}$) [6], whereas HIIT or AIT is often used in the context of exercise intensities between 80 % and 90 % $\overset{.}{\mathrm{V}}{\mathrm{O}}_{2\mathrm{peak}}$ [3, 7, 8].

Acute microvascular responses to exercise (e.g., enhanced blood flow and local blood supply of small vessels in active tissue [9, 10]) have also been reported as long-term adaptations following HIT (e.g., by increased vasodilatory capacity [9] and augmented capillarization [5, 11]). Fu et al. showed these effects to be superior to those of moderate continuous training in patients with heart failure [7]. Furthermore, several studies have suggested that aerobic adaptations are facilitated by the above-mentioned microvascular mechanisms following HIIT/AIT [3, 4, 7].

Although the literature contains substantial knowledge on the acute effects of exercise on local muscle perfusion in general [10, 12–15], the acute post-exercise effects of different exercise durations on local muscle oxygen availability and blood supply have not yet been sufficiently examined. Most studies have focused on the influence of exercise intensity [16–19] or examined the effect of complete training sessions with only two different work-interval durations [20].

Relative changes in local, total hemoglobin concentration (ΔTHb) is a widely used parameter for blood-volume changes in terms of capillary filling [21–23] and vasodilation [24] and can be noninvasively monitored using near-infrared spectroscopy (NIRS) [22]. With continuous-wave spectrometers, relative changes in the THb and deoxygenated (HHb) and oxygenated Hb (O_2_Hb) concentrations can be observed because of the distinct relative transparency of HHb and O_2_Hb for specific, near-infrared light wavelengths.

The potential of exercise to prolong augmentation of the local blood supply could be an important determinant for metabolic adaptations. The aim of our study was to identify the association between durations of HIIT/AIT exercise bouts and relative changes in post-exercise concentrations of THb, O_2_Hb, and HHb to examine (1) the post-exercise blood supply and (2) local oxygen availability.

## Methods

### Subjects

A total of 18 healthy, physically active males volunteered to participate in the present study (see Table 1). Participants exercised at least 2 times per week in various sports, whereas aerobic exercise contributed an average of 4 ± 3 h per week. The inclusion criteria included age (18–35 years) and a physically active background. Exclusions included acute or chronic diseases (such as chronic heart disease), diabetes type II, epilepsy, and relevant diseases of the liver and kidney, as well as smoking and excessive alcohol consumption. All subjects were familiar with cycling exercise and exhaustive exercise testing. They received detailed information on the purpose and procedures of the study and their written informed consent was obtained. The local Ethics Committee of the Technische Universität München approved the study protocol (#67/14).

**Table 1:** Subject data

Parameter	
N	18
Age (years)	28 ± 5
Weight (kg)	77.7 ± 4.9
Height (cm)	181.3 ± 5.2
Skinfold thickness (mm)	7.8 ± 2.9
$\overset{.}{\mathrm{V}}{\mathrm{O}}_{2\mathrm{peak}}$ (ml·kg^−1^·min^−1^)	50.4 ± 5.7
GET (% $\overset{.}{\mathrm{V}}{\mathrm{O}}_{2\mathrm{peak}}$)	58 ± 13
n GET60+	7
n GET60−	11

Values are expressed as mean ± SD

### Test design and procedures

Subjects reported to the laboratory for two sessions. We conducted preliminary tests (see section *preliminary maximal testing*) during the first session. During the second session, we aimed to examine the local blood supply in relation to exercise duration. Both sessions had to be completed within one week and had to be separated by at least 48 h to enable full recovery. The subjects were instructed not to perform exhaustive activity 24 h before each session and to avoid caffeinated and high-carbohydrate beverages 2 h before each session. Special bicycle shoes and pedals were not allowed during the ergometer tests to reduce the influence of cycling technique.

### Preliminary maximal testing

During the first visit, all subjects performed an incremental test to exhaustion on an electrically braked cycle ergometer (Lode Excalibur; Lode, Groningen, The Netherlands) to determine $\overset{.}{\mathrm{V}}{\mathrm{O}}_{2\mathrm{peak}}$ and gas-exchange threshold (GET), according to the v-slope method [25]. The 60-W load was increased by 10 W every 30 s and led to volitional exhaustion within 14.8 ± 1.5 min, which has been reported as a suitable time span for reaching maximum aerobic contribution [26]. Subjects were allowed to individually choose the cadence during the cycle ergometer trials but were asked to maintain a minimum cadence of >70 revolutions/min. The test was terminated when pedaling frequency could no longer be maintained. Although all subjects were physically active, healthy, and familiar with exhaustive exercise, $\overset{.}{\mathrm{V}}{\mathrm{O}}_2$ did not attain a plateau in two subjects. Thus, we measured $\overset{.}{\mathrm{V}}{\mathrm{O}}_{2\mathrm{peak}}$ instead of maximum oxygen uptake $\left({\overset{.}{\mathrm{V}}\mathrm{O}}_{2 \max}\right)$. However, Day et al. showed that $\overset{.}{\mathrm{V}}{\mathrm{O}}_{2\mathrm{peak}}$ and ${\overset{.}{\mathrm{V}}\mathrm{O}}_{2 \max }$ do not differ in a healthy, physically active population when maximum effort is put forth [27]. To ensure maximum effort, we verbally motivated subjects at the end of the test and datasets were included only if a maximum respiratory exchange ratio >1.1 had been attained.

### Experimental testing

Subjects were asked to complete a randomized protocol consisting of five exercise bouts with different durations (30, 60, 90, 120, and 240 s), separated by 5-min passive, recovery periods. Exercise intensity was determined corresponding to 80 % $\overset{.}{\mathrm{V}}{\mathrm{O}}_{2\mathrm{peak}}$_,_ based on the data of the preliminary maximal test. Consequently, $\overset{.}{\mathrm{V}}{\mathrm{O}}_2$ did not attain 80 % during the 30-s and 60-s exercise bouts because of the finite $\overset{.}{\mathrm{V}}{\mathrm{O}}_2$ -kinetics during exercise on transitions [28]. On the other hand, $\overset{.}{\mathrm{V}}{\mathrm{O}}_2$ exceeded 80 % slightly during the 240-s bout because of the slow component of $\overset{.}{\mathrm{V}}{\mathrm{O}}_2$ [28] (Table 2). The exercise bouts were randomly assigned. It is worth pointing out that Buchheit et al. used 3-min recovery periods for similar measurements [29]. However, we noted that the recovery times of HHb and O_2_Hb exceeded 180 s (Table 2), so we decided to use 5-min recovery periods instead. The applied exercise durations, which are commonly used in HIIT/AIT prescriptions, represent interval bouts that range from short to long duration [1]. The exercise intensity of 80 % $\overset{.}{\mathrm{V}}{\mathrm{O}}_{2\mathrm{peak}}$ is commonly used for HIIT/AIT in combination with long-duration intervals, while short-exercise bouts up to 60 s are associated with supramaximal work rates [3]. In spite of that, a shorter interval (30 s) was included in this setup to obtain a detailed overview on the relationship of exercise duration and post-exercise blood supply.

**Table 2:** Recovery parameters for ΔO2Hb, ΔHHb, and ΔTHb

Exercise duration	30 s	60 s	90 s	120 s	240 s
OS_THb (Δμmol^**.**^l^−1^)	3.05 ± 1.26	6.00 ± 2.78	6.97 ± 2.11	6.98 ± 2.30	7.25 ± 3.03
OS_O_2_Hb (Δμmol^**.**^l^−1^)	1.09 ± 1.32	4.79 ± 2.37	6.34 ± 1.88	6.54 ± 2.90	5.98 ± 2.75
GET60+: OS_O_2_Hb (Δμmol^**.**^l^−1^)	1.14 ± 1.60	6.00 ± 2.70	7.02 ± 2.11	--	6.08 ± 2.15
GET60−: OS_O_2_Hb (Δμmol^**.**^l^−1^)	1.06 ± 1.19	4.02 ± 1.88	5.91 ± 1.67	--	5.91 ± 3.18
Recovery of ΔTHb (s)	198 ± 86	206 ± 82	199 ± 65	210 ± 57	234 ± 37
Recovery of ΔO_2_Hb (s)	243 ± 56	222 ± 66	239 ± 23	195 ± 71	227 ± 56
Recovery of ΔHHb (s)	212 ± 88	210 ± 76	206 ± 79	185 ± 84	243 ± 31
SD_REC_ ΔTHb (Δμmol^**.**^l^−1^)	0.18 ± 0.21	0.36 ± 0.27	0.24 ± 0.12	0.21 ± 0.14	0.26 ± 1.23
SD_REC_ ΔO_2_Hb (Δμmol^**.**^l^−1^)	0.19 ± 0.13	0.34 ± 0.28	0.34 ± 0.12	0.20 ± 0.09	0.28 ± 0.25
SD_REC_ ΔHHb (Δμmol^**.**^l^−1^)	0.11 ± 0.09	0.20 ± 0.09	0.11 ± 0.05	0.15 ± 0.09	0.12 ± 0.04
Time-to-peak ΔTHb (s)	57 ± 29	65 ± 50	79 ± 57	71 ± 37	72 ± 44
Time-to-peak ΔO_2_Hb (s)	151 ± 81*	103 ± 59	81 ± 26	91 ± 48	94 ± 39
End-exercise % $\overset{.}{\mathrm{V}}{\mathrm{O}}_2$ (%)	43 ± 8*	73 ± 5	81 ± 6	82 ± 6	86 ± 5

*n* = 18 (except GET60+/−); values are expressed as mean ± SD; Significant results for OS_THb/O_2_Hb are presented in Fig. 4; SD_REC_ΔTHb/O_2_Hb/HHb represent the average standard deviation of the last 30 s of recovery; Time-to-peak values indicate the time from cessation of exercise to the highest value reached during the subsequent recovery period (asterisks indicate significant higher values compared to the other values, ** *P* < 0.01); End-exercise % $\overset{.}{\mathrm{V}}{\mathrm{O}}_2$ values are averaged values obtained from the final 10 s of each bout

A 3-min baseline measurement (BASE_0_) was conducted prior to the first interval with the subject sitting passively on the ergometer. This measurement was followed by a warm-up, consisting of 1 min at 60 % $\overset{.}{\mathrm{V}}{\mathrm{O}}_{2\mathrm{peak}}$ and 3 min at 80 % $\overset{.}{\mathrm{V}}{\mathrm{O}}_{2\mathrm{peak}}$. Prior observations revealed that O_2_Hb levels would be markedly elevated, following the first exercise bout but would attain similar values between subsequent exercise bouts, which could be due to increased skin blood flow [30]. Furthermore, pre-exercise affects the speed of aerobic metabolism during subsequent exercise on-transitions [31]. Hence, pre-exercise was used to activate muscle blood flow and metabolism to avoid order effects for the first experimental condition. During BASE_0_, as well as during recovery periods, subjects had to maintain a standardized position, keeping the right foot on a bar between the pedals to minimize movement artifacts by unwanted muscular activity on the NIRS signal.

**Fig. 1:**
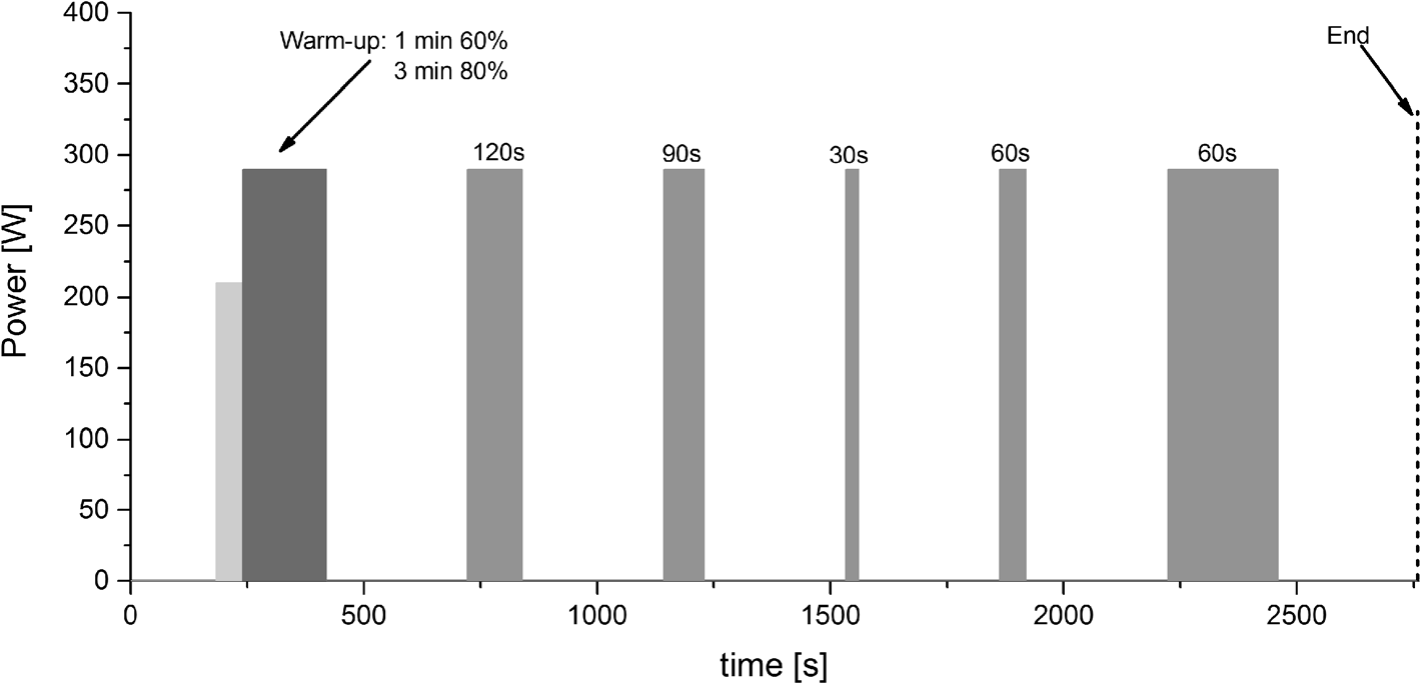
Scheme of an exemplary experimental protocol. Subjects had to maintain a standardized recovery position, sitting on the ergometer during baseline measurement prior to the warm-up and recovery periods between exercise bouts

An exemplary scheme of the experimental protocol is displayed in Fig. 1.

### Gas exchange and heart rate measurement

Heart rate was monitored (Polar Team^2,^ Polar Electro, Kempele, Finland) throughout all sessions and respiratory parameters were measured continuously with a breath-by-breath metabolic cart (ZAN 600USB; Nspire Health, Oberthulba, Germany), which had been used and validated previously [32, 33]. The oxygen-uptake $\left({\overset{.}{\mathrm{V}}\mathrm{O}}_2\right)$ curve was smoothed with a 30-s moving average and heart-rate data were smoothed with a 5-s moving average. $\overset{.}{\mathrm{V}}{\mathrm{O}}_{2\mathrm{peak}}$ was determined as the highest value reached during exercise and was expressed relative to body weight (ml·kg^−1^·min^−1^). GET was determined using the v-slope method [25] and was confirmed with ventilatory equivalents for O_2_ and CO_2_. Peak heart rate was recorded for each exercise bout.

### NIRS

A wireless continuous-wave NIRS device (PortaMon, Artinis Medical Systems, Zetten, The Netherlands) was used to measure local changes in Hb concentration in the right, vastus lateralis muscle during all sessions. NIRS data were collected using the software Oxysoft (Artinis Medical Systems) and were sampled with a frequency of 10 Hz and expressed as Δμmol^**.**^L^−1^. The PortaMon probe consists of three light sources (wavelengths: 760 nm and 850 nm) separated by 3, 3.5, and 4 cm from the receiving optode, while only data measured with the 3.5 cm optode distance were taken into account, corresponding to a penetration depth of approximately 1.75 cm [17, 34]. The probe, which was firmly attached with adhesive tape to the shaved skin above the distal part of the right vastus lateralis muscle, was covered with a lightproof cloth to prevent any influences of ambient light. The position of the probe was marked with a surgical marker to detect possible shifts (of the probe) during the test.

The PortaMon probe simultaneously uses the modified Lambert–Beer law and spatially resolved spectroscopy [22], which enables the observation of relative changes in O_2_Hb, HHb, and THb. Because the exact path-length of the photons which penetrate the tissue is unknown when using continuous-wave spectroscopy, a differential path-length factor has to be used [22]. In the present study, we used a differential path-length factor of 4, which has been applied by other studies in measurements of muscle tissue [29, 34]. However, we are aware of the uncertainty of the differential path-length factor for muscle tissue and, hence, have reported all changes relative to individually measured baseline values (ΔTHb/O2Hb/HHb). Due to the equal absorption properties of Hb and myoglobin (Mb), it is not possible to distinguish between their contributions on the NIRS signal. However, the contribution of Mb to the NIRS signal is assumed to be minimal [22]. For simplification, “Hb + Mb” are termed “Hb” in the present study.

**Fig. 2:**
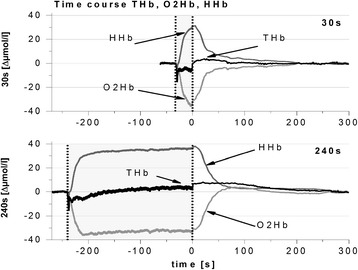
Typical kinetics of ΔO_2_Hb, ΔHHb, and ΔTHb during and after 30-s and 240-s cycling at 80 % $\overset{.}{\mathrm{V}}{\mathrm{O}}_{2\mathrm{peak}}$ in relation to baseline values (set to zero). Note the marked overshoot in ΔO_2_Hb following the 240-s exercise interval compared to the 30-s interval

After collection, the data were smoothed with a 1-s moving average. The data of the last 30 s of BASE_0_ were averaged. Baseline values were measured again for each exercise bout in the last 30 s of the previous recovery period (BASE_30–240_). The recovery time of ΔTHb/O2Hb/HHb was determined by assessing the time elapsed from cessation of exercise and the point where the value reached the average of the last 30 s of recovery ±5 standard deviations for the first time (Table 2). We chose five standard deviations because the behavior of the NIRS parameters was not completely stable, even in the end of each recovery period, due to subjects’ involuntary movements. End-exercise values (averaged over the last 10 s of exercise) were assessed and expressed in relation to the respective baseline values for each exercise bout (EE_O2Hb/HHb/THb). The early post-exercise overcompensation appears as an overshoot (OS) of ΔO_2_Hb and ΔTHb after termination of exercise and was also expressed in relation to baseline values (Fig. 2), as well as the time-to-peak from cessation of exercise (Table 2). To control possible changes of BASE, we calculated the difference between the baseline before and after each exercise bout (ΔBASE40–90).

### Statistical analyses

Statistical analyses were performed using the statistical software package, SPSS version 20 (SPSS Inc., Chicago, IL, USA). We used repeated measures analysis of variance (ANOVA) to detect possible differences in the effect of the various exercise durations on OS_O_2_Hb/THb, end-exercise concentrations of ΔO_2_Hb/ΔHHb/ΔTHb, peak heart rate, time-to-peak for ΔO_2_Hb, and ΔTHb, as well as ΔBASE for ΔO_2_Hb/ΔHHb/ΔTHb. Data were tested for sphericity with Mauchly’s test and were corrected using the Greenhouse–Geisser method [35] when necessary. If ANOVA showed a significant main effect, pairwise comparisons were performed with Bonferroni–Holm corrected post-hoc tests. For OS_O_2_Hb and OS_THb, the relative aerobic fitness was considered a group factor, whereby subjects with GET below 60 % $\overset{.}{\mathrm{V}}{\mathrm{O}}_{2\mathrm{peak}}$ and above 60 % $\overset{.}{\mathrm{V}}{\mathrm{O}}_{2\mathrm{peak}}$ were assigned as “GET60−” and “GET60+”, respectively. The cut-off point was chosen as 60 % $\overset{.}{\mathrm{V}}{\mathrm{O}}_{2\mathrm{peak}}$ because this approximately corresponds to the mean GET for the entire sample (Table 1). Separate ANOVAs were performed for each level of aerobic fitness when a significant interaction of *time × aerobic fitness* could be observed, whereas the differences between 30, 60, 90, and 240 s of exercise were focused on. “Pre-exercise” values of ΔO_2_Hb and ΔTHb were individually compared with the corresponding post-exercise peak values (which have been used to calculate OS) using *t*-tests. The purpose of the individual comparisons was to verify whether the post-exercise elevation in both parameters was significant. The same procedure was conducted to compare baseline values before and after particular exercise bouts to test for possible baseline drifts. A *P* value of ≤0.05 was considered statistically significant.

## Results

### Parameters during exercise

**Fig. 3:**
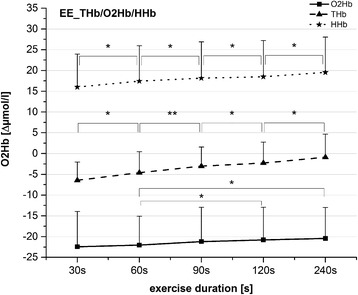
End-exercise (EE) values (mean ± SD) of ΔO2Hb (*solid line*), ΔHHb (*dotted line*), and ΔTHb (*broken line*). *Asterisks* represent results from post-hoc tests in relation to values marked with double lines; * = *P* < 0.05; ** = *P* < 0.01; *** = *P* < 0.001

Figure 2 shows a representative time course of one subject showing post-exercise ΔHHb, ΔO_2_Hb, and ΔTHb kinetics during and following 30-s to 240-s exercise bouts at 80 % $\overset{.}{\mathrm{V}}{\mathrm{O}}_{2\mathrm{peak}}$. An ANOVA revealed a significant effect of the factor *time* on end-exercise ΔO_2_Hb (*F*_*4,68*_ = 4.1; *P* = 0.005), ΔHHb (*F*_*3,54*_ = 22.5; *P* < 0.001), and ΔTHb values (*F*_*3,42*_ = 24.7; *P* < 0.001, Fig. 3). For ΔO2Hb, post hoc tests showed significantly lower values at the end of the 60-s exercise bouts than in the 120-s and 240-s exercise bouts (*P* ≤ 0.010). The end-exercise ΔTHb values increased significantly with increasing exercise duration (*P* ≤ 0.042). End-exercise ΔHHb showed a similar time course (i.e.*,* one that increased significantly with increasing exercise duration [*P* ≤ 0.046]), except when the value at 90 s was compared with that at 120 s.

**Fig. 4:**
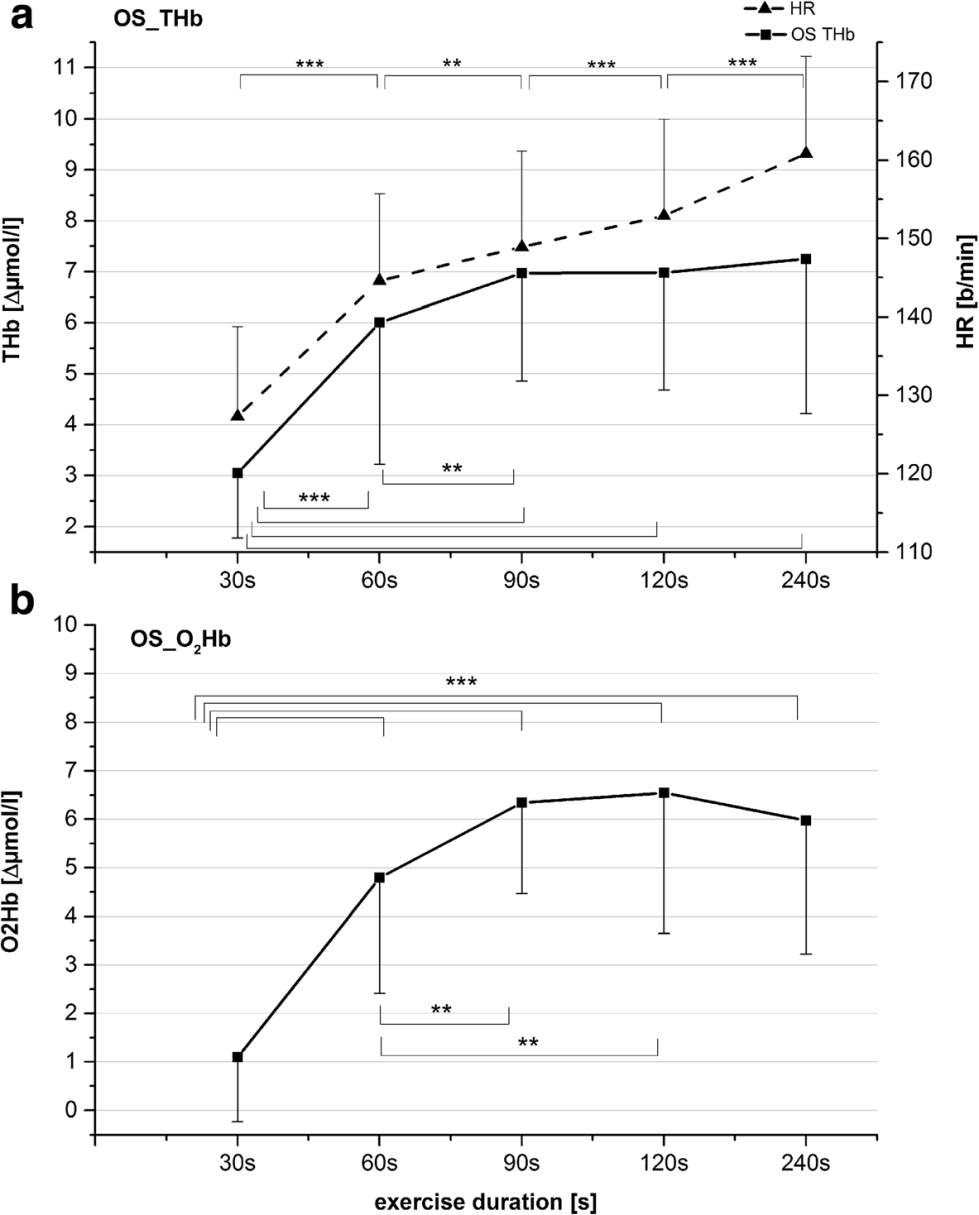
**a** Overshoot values of ΔTHb (*solid line*) and end-exercise (EE) heart rate values (*broken line*) (mean ± SD) after the five exercise bouts. **b** Overshoot values of ΔO2Hb (mean ± SD) after five exercise bouts. *Asterisks* represent results from post-hoc tests in relation to values marked with double lines; * *P* < 0.05; ** *P* < 0.01; *** *P* < 0.001. Heart-rate values all differed significantly from each other at *P* < 0.001

Heart-rate values were also affected by exercise duration (*F*_*3,41*_ = 116; *P* < 0.001) and increased significantly with this factor (*P* < 0.001, Fig. 4a).

### Post-exercise parameters

Immediately after cessation of the exercise bouts, ΔO_2_Hb, ΔHHb, and (as a consequence) ΔTHb increased abruptly due to the relaxation of the working muscle. Afterward, ΔHHb decreased in an exponential manner and reached a stable level without showing any clear and systematic undershoot. ΔO_2_Hb increased inversely following 30 s of exercise. Following 60, 90, 120, and 240 s, of exercising at 80 % $\overset{.}{\mathrm{V}}{\mathrm{O}}_{2\mathrm{peak}}$, the ΔO_2_Hb increased abruptly and reached an overshoot (OS_O_2_Hb), relative to the pre-exercise values (*P* < 0.001) within 80 s. This time-to-peak was significantly longer following 30-s of exercise (ANOVA: *F*_*4,68*_ = 6.0; *P* = 0.008; post-hoc: *P* ≤ 0.018, see Table 2). Subsequently, ΔO_2_Hb decreased to baseline level. Although there was no overcompensation with a subsequent decrease in ΔO_2_Hb after 30-s exercise (Fig. 2), the highest post-exercise value (which actually occurred in the end of the recovery period) was determined as OS_O_2_Hb_30_. Because there were small increases in baseline values throughout the trial, the overshoot following 30 s was also significantly higher (*P* = 0.003) than the pre-exercise baseline values (changes in baseline values were not related to exercise duration). The post-exercise overcompensation also occurred for ΔTHb after all exercise bouts (*P* < 0.001), whereas time-to-peak was not significantly related to exercise duration (Table 2).

ANOVA showed significant effects of the factor *time* on OS_O_2_Hb (*F*_*3,53*_ = 26.6; *P* < 0.001) and OS_THb (*F*_*2,40*_ = 23.2; *P* < 0.001). Based on post-hoc tests, the OS_O_2_Hb increased with increasing length of the exercise bouts up to 90-s exercise duration (*P* ≤ 0.005) (Fig. 4b). Also, OS_O_2_Hb values following the 120-s and 240-s exercise bouts were not significantly different from those following the 60-s bout.

OS_THb had a similar time course to OS_O_2_Hb. OS_THb_60_ was significantly higher (*P* ≤ 0.005) than OS_THb_30_ and was significantly lower than OS_THb_120_ (*P* ≤ 0.008). However, OS_THb_90_ was not different from OS_THb_60_ and OS_THb_120_ (Fig. 4a). OS_THb_240_ was not different from OS_THb_120_.

**Fig. 5:**
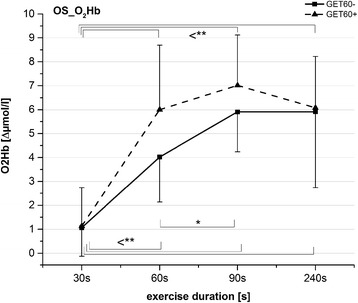
Overshoot values of ΔO2Hb (mean ± SD) for GET60+ and GET60−. *Asterisks* represent results from post-hoc tests in relation to values marked with double lines; *P < 0.05; ** *P* < 0.01; *** *P* < 0.001

We noted a significant interaction of *time × aerobic fitness* in OS_O_2_Hb (*F*_*3,51*_ = 4.1; *P* = 0.011). In the GET60+ group, OS_O_2_Hb was significantly influenced by the factor *time* (*F*_*3,18*_ = 17.3; *P* < 0.001). Post hoc tests showed significantly lower values for OS_O_2_Hb following 30 s of exercise than those for the other exercise intensities (*P* ≤ 0.007). The factor *time* also affected OS_O_2_Hb in the GET60− group (*F*_*3,30*_ = 16.5; *P* < 0.001), but contrary to the GET60+ group, this parameter increased progressively from 30 s to 90 s in exercise duration (*P* ≤ 0.015) and did not change significantly thereafter (Fig. 5).

However, no significant interaction of *time × aerobic fitness* could be found for OS_THb (*F*_*3,34*_ = 2.9; *P* = 0.068).

All parameters (ΔO_2_Hb, ΔHHb, and ΔTHb) reached stable values at the end of each recovery period (which was defined as the new baseline for the subsequent exercise bout). These new baseline values did not show any significant exercise “duration-related” differences; neither when sorted by exercise intensity nor by chronological order of the exercise bouts (Table 3).

**Table 3:** Baseline drifts

A. Exercise duration	ANOVA	30 s	60 s	90 s	120 s	240 s
ΔBASE_THb (Δμmol^**.**^l^−1^)	*P =* 0.182	0.79 ± 1.18	0.76 ± 2.84	1.06 ± 1.26	1.17 ± 0.98	0.83 ± 1.14
ΔBASE_O_2_Hb (Δμmol^**.**^l^−1^)	*P =* 0.069	−1.54 ± 1.98	−0.62 ± 4.37	0.95 ± 2.31	1.95 ± 1.64	0.65 ± 1.00
ΔBASE_HHb (Δμmol^**.**^l^−1^)	*P =* 0.086	0.97 ± 1.24	0.51 ± 2.01	−0.09 ± 1.22	−0.41 ± 0.75	0.72 ± 1.52
B. No. of exercise bout		1	2	3	4	5
ΔBASE_THb (Δμmol^**.**^l^−1^)	*P =* 0.564	0.87 ± 3.05	1.16 ± 1.13	0.34 ± 1.24	1.34 ± 1.20	0.17 ± 1.29
ΔBASE_O_2_Hb (Δμmol^**.**^l^−1^)	*P =* 0.271	0.18 ± 3.30	1.14 ± 1.82	0.77 ± 1.69	1.76 ± 1.75	−0.87 ± 2.43
ΔBASE_HHb (Δμmol^**.**^l^−1^)	*P =* 0.218	0.69 ± 1.40	−0.20 ± 1.23	−0.43 ± 0.63	−0.42 ± 1.54	1.04 ± 1.34

*n* = 18; values are expressed as mean ± SD; ΔBASE values are the differences between the baseline values before and after the exercise bouts; Panel A shows baseline drifts sorted by exercise duration while panel B shows baseline drifts sorted by chronological order of the exercise bouts

## Discussion

The data of the present study show that cycling exercise at 80 % $\overset{.}{\mathrm{V}}{\mathrm{O}}_{2\mathrm{peak}}$ triggers post-exercise hyperemia, which is indicated by an overshoot in ΔTHb following exercise bouts in relation to pre-exercise values. ΔTHb has been used as an indirect measure for blood volume previously [21–23]. When we considered the five different exercise durations, the 30-s and 60-s exercise bouts evoked significantly lower overshoot values than the longer exercise bouts.

Because the relationship of post-exercise overshoots of ΔTHb and ΔO_2_Hb evolved similarly, we suggested that the arterial blood supply primarily accounts for the increased post-exercise ΔTHb. A reduced outflow would be expected to emerge as a concomitant increase in post-exercise ΔHHb at the point of OS. Like OS_THb, OS_O_2_Hb increased stepwise from 30 to 60-s and from 60 to 90-s exercise, indicating an increasing availability of oxygen in the area of interrogation up to exercise durations ≥90 s.

To the best of our knowledge, the present study is the first to investigate post-exercise muscle reoxygenation in relation to single exercise bouts of different durations as they are used in interval-training regimens. In general, a number of studies have reported on post-exercise hyperemia following single exercise bouts of similar durations as they were applied in the present study [16, 17, 19]. Furthermore, Danduran et al. [18] observed hyperemia using NIRS following a graded exercise test design in healthy children. Post-exercise reoxygenation was analyzed in various studies. A significant dependency of post-exercise Hb and muscle oxygenation recovery kinetics on exercise intensity (in terms of a forced reoxygenation following higher exercise intensities) has been reported previously [16, 17, 19]. Despite this dependence, reoxygenation time is apparently not influenced by exercise intensity [19, 29]. When Belfry et al. [36] compared interval-training regimens with constant-load exercise at equal intensity, they found a better matching of O_2_ delivery to O_2_ utilization during exercise for the interval regimens than that in constant-load exercise. O_2_ delivery during interval training was enhanced when recovery was applied in the moderate intensity domain in comparison to “low-intensity” active recovery. Besides the muscle-pump effect, which was presumably increased during interval training with “moderate intensity” recovery, the authors suggested enhanced local vasodilation to be an important determinant for those results.

Zafeiridis et al. [20] were also able to find improved local oxygen delivery during exercise. In contrast to Belfry et al., they found no differences between constant-load exercise and two interval-training regimens with different work-interval durations. The different results in these two studies could likely be attributed to differences in the two study protocols. As we did in the present study, Zafeiridis et al. examined the effect of different work-interval durations. They used work intervals of 30 s with an intensity of 110 % of the power output corresponding to $\overset{.}{\mathrm{V}}{\mathrm{O}}_{2 \max }$ and 120-s intervals with an intensity of 95 % $\overset{.}{\mathrm{V}}{\mathrm{O}}_{2 \max }$ and did not find significantly different local-oxygen delivery during both interval-training regimens. This finding of Zafeiridis et al. is contrary to our findings, which showed significantly higher post-exercise oxygen availability and blood supply following 120 s of exercise than that following 30 s of exercise with 80 % $\overset{.}{\mathrm{V}}{\mathrm{O}}_{2\mathrm{peak}}$. We cite two possible explanations for this difference. First, the dependency of post-exercise blood supply and oxygen availability on exercise duration, as it is shown in the present study, appears to be valid only within a certain exercise intensity range. In a previous study, we examined the effect of exercise intensity on post-exercise blood supply and oxygen availability and observed a non-linear relationship [19]. We found no differences between 80 and 90 % $\overset{.}{\mathrm{V}}{\mathrm{O}}_{2\mathrm{peak}}$, but did not include higher exercise intensities; therefore, it might be possible that our results are not valid for supramaximal exercise. Second, Zafeiridis et al. used different recovery durations between work intervals, which could have contributed to different results. It could be that 30-s work intervals cause hyperemia similar to that associated with 120-s intervals, when recovery periods are adjusted appropriately.

Although the methods used in our study do not provide sufficiently detailed information on the complex regulation of local muscle perfusion, we call attention to a few possible explanations for our results. In general, cardiac output and artery flow increase as a response to dynamic exercise [12]. In exercising muscles, two antagonistic mechanisms happen: There is global sympathetic mediated vasoconstriction on the one hand whereas acute vasodilation occurs in active tissue secondary to a promoted release of vasoactive substances on the other hand. Because vasodilation superimposes sympathetic mediated vasoconstriction in active muscles, capillary perfusion is increased [10, 37, 38]. This process is supported mechanically by rhythmic contractions of the muscle during cycle exercise (muscle pump) [12].

Previous research has shown a biphasic response of muscle blood flow to exercise with an early, rapid response within the first 5–7 s and a prolonged, secondary response starting 15–20 s from the onset of exercise [15], while a steady state is reached within 1–3 min. Hence, blood-flow adjustments to exercise occur rather quickly. After termination of exercise, blood-flow recovery has been reported to be slower following heavy exercise than following moderate exercise [13]. This slowing of blood-flow recovery is most likely due to the prolonged presence of vasoactive substances. In rats, it had been shown that acute, vasodilatory responses to exercise can last up to two days or more [39]. Consequently, prolonged, local vasodilation seems to be dependent on the extent of the release of vasoactive substances during the previous exercise bout.

But which mechanisms are crucial for the increased post-exercise oxygen availability between 30 and 90 s of exercise at 80 % $\overset{.}{\mathrm{V}}{\mathrm{O}}_{2\mathrm{peak}}$? We did not measure cardiac output in this study, but our data reveals some basis for speculation. The local oxygen availability was equal from 90 to 240 s of exercise duration (Fig. 4b), while end-exercise heart rate increased continuously. We assumed accordingly that end-exercise cardiac output also increased continuously with exercise duration (otherwise, stroke volume would have declined, which is unlikely). Hence, if the prolonged recovery of cardiac output would have greater implications on post-exercise hyperemia, then post-exercise hyperemia would have been expected to increase continuously following exercise durations >90 s. In a previous study, we observed similar characteristics in the relationship of local blood supply and exercise intensity [19]. Among local mechanisms, the muscle pump is neglectable as an explanation of our results as it stops with cessation of exercise, which leads to an abrupt drop in muscle blood flow [13].

Local Vasodilation remains as the most plausible determinant for the hyperemia and increased post-exercise oxygen availability following cycling exercise ≥90 s at 80 % $\overset{.}{\mathrm{V}}{\mathrm{O}}_{2\mathrm{peak}}$. Factors that trigger vasodilation are various. Among those factors, chemically and flow mediated vasodilation have to be highlighted when discussing exercise induced vasodilation [40]. In particular, adenosine, acetylcholine, and shear stress are presumed to trigger exercise-induced effects on vasodilation [41, 42]. It can be assumed that blood flow during exercise and prolonged vasodilation of small vessels in the post-exercise phase are positively related. It is likely that this effect was more pronounced (in our study) following exercise durations ≥90 s compared to shorter exercise bouts. And again, because we did not measure vasodilating substances or cardiac output and blood flow velocity directly, the implications of the above-mentioned mechanisms for our study results remain speculation-based.

We have shown that oxygen availability following cycling exercise is dependent on exercise duration. Our results demonstrate that this dependency is influenced by the relative aerobic fitness (i.e.*,* GET), expressed as a percentage of $\overset{.}{\mathrm{V}}{\mathrm{O}}_{2\mathrm{peak}}$. In the GET60+ group, 60 s of exercise were sufficient to evoke a post-exercise overshoot of ΔO_2_Hb, which was equal to the overshoots following longer exercise bouts. In the GET60− group, 90 s of exercise were needed to obtain those high values. Hence, adjustments in local vasodilation are possibly faster in subjects showing high relative aerobic power. This means that in subjects with higher aerobic fitness, exercise durations of 60 s are sufficient to evoke an increased post-exercise hyperemia and increased oxygen availability.

### Advantages of improved post-exercise O_2_ availability

There are several advantages of improved local blood and oxygen supply, as it has been observed following exercise durations >90 s at 80 % $\overset{.}{\mathrm{V}}{\mathrm{O}}_{2\mathrm{peak}}$. First, gas exchange is facilitated when the functional cross-sectional area of capillaries is increased [9]. Moreover, muscle reoxygenation has been shown to improve with endurance training [43]. A higher local muscle perfusion is associated with enhanced oxidative metabolism, such as fatty acid oxidation [44–46]. Romijn et al. [44] showed reduced fatty acid mobilization during strenuous exercise (85 % $\overset{.}{\mathrm{V}}{\mathrm{O}}_{2\mathrm{peak}}$), as well as a strongly increased post-exercise plasma fatty-acid availability following strenuous exercise [44, 45]. This increased post-exercise, plasma fatty-acid availability may be one explanation for improvements in fatty-acid oxidation capacity as long-term metabolic adaptations that have been observed following HIT in women [3]. Kimber et al. [47] found a significant dependency of post-exercise fatty-acid oxidation on fatty-acid availability. Exercise that increases fatty-acid availability is, therefore, likely to enhance fatty-acid oxidation after or between exercise bouts. The improved post-exercise blood supply during recovery or, when active recovery is applied, during the relief interval could therefore augment fatty-acid oxidation in particular. However, we reiterate that these suggestions are speculative because we did not measure fatty-acid oxidation.

### Study limitations

When using NIRS, the influence of adipose tissue thickness (ATT) has to be considered [34]. If skin and subcutaneous tissue thickness is near to or exceeds the penetration depth of the emitted photons, muscular effects will be blunted. Skin and subcutaneous thickness was approximately 3.75 mm (7.5 ± 3.1 mm skinfold thickness, measured with a caliper). Because penetration depth has been shown to be approximately half of the optode distance [22, 34], it can be concluded that NIRS signal was capable for measuring muscle oxidation in our study as the inter-optode distance was 3.5 cm, enabling a penetration depth of 1.75 cm.

Another point to mention is the 5-min recovery period in-between exercise bouts, which was too short to enable a full recovery of ΔO_2_Hb and ΔTHb. It therefore has to be considered, that prior exercise bouts influenced the subsequent exercise bouts [31] First, this baseline drift in ΔO_2_Hb and ΔTHb did not show any relationship to exercise duration. Second, we randomized the order of the exercise bouts and placed an intensive warm-up in front of the first experimental exercise bout. Consequently, this issue should not have influenced our results.

We recruited only male subjects to reduce variability in subject characteristics and because of the minor adipose tissue thickness in males relative to that in females, which apparently affects NIRS signals [34]. Hence, the relevance of our results on females is limited.

Work intervals within interval-training regimens are described by interval duration and interval intensity. We investigated the influence of exercise intensity on local muscle O_2_ availability and blood supply in an earlier study [19]. The present study aimed to examine the effect of duration, using isolated, “interval-training-associated” exercise bouts. But besides the work interval, several other variables in interval-training prescriptions, such as intensity and duration of the recovery interval and number of repetitions, should be noted. The latter is of particular importance. Because HIT consists of repeated sets of transitions from high to low intensity, work-interval durations <90 s also are potentially effective to increase post-exercise oxygen availability due to a cumulative effect. This “repeated bout” effect could occur when low intervals are too short to provide an adequate recovery. It is therefore conceivable that effects of HIT on local oxygen availability could be similar across different exercise durations, for example when the work-to-rest ratio is kept constant. It has to be highlighted that all intervals in our study had been carried out with the same exercise intensity. Usually, work intervals are prescribed with higher intensity during “short-interval” HIT-regiments [1]. As a consequence, the low interval has to be extended to provide sufficient recovery to enable the completion of the following work interval. Consequently, the relationship between interval intensity, duration and recovery has to be focused in future research.

Finally, the post-exercise O_2_ availability and blood supply are two of several determinants causing aerobic adaptations. Investigation of other determinants may require other, optimal “work-interval” durations.

## Conclusion

Exercise duration in HIT has a significant impact on post-exercise oxygen availability following cycling exercise with 80 % $\overset{.}{\mathrm{V}}{\mathrm{O}}_{2\mathrm{peak}}$. The highest post-exercise oxygen availability is induced with exercise durations of 90 s and longer, whereas no further increase is evoked by longer exercise durations up to 240 s. Post-exercise oxygen availability is dependent on aerobic fitness. Subjects, who reached GET below 60 % $\overset{.}{\mathrm{V}}{\mathrm{O}}_{2\mathrm{peak}}$ needed 90 s to reach the maximum post-exercise oxygen availability, while subjects who reached GET above 60 % $\overset{.}{\mathrm{V}}{\mathrm{O}}_{2\mathrm{peak}}$ reached the maximum post-exercise oxygen availability following 60 s exercise. To facilitate oxidative adaptations, work intervals within interval-training regimens should be prescribed with a minimum length of 60 s, depending on aerobic fitness, whereas it has to be reiterated that the transferability of our findings to exercise intensities that are different to 80 % $\overset{.}{\mathrm{V}}{\mathrm{O}}_{2\mathrm{peak}}$ may be limited.

### Ethics approval and consent to participate

The study protocol was approved by the local Ethics Committee of the Technische Universität München, Klinikum Rechts der Isar (#67/14). All subjects received detailed information on the purpose and procedures of the study and gave their written informed consent.

### Consent for publications

Not applicable.

### Availability of data and materials

The dataset supporting the conclusions of this article is available in the Open Science Framework repository, you can access the dataset by typing following address in your browser: osf.io/5hq2g.

## Abbreviations

AIT: aerobic interval training
ANOVA: analysis of variance
ATT: adipose tissue thickness
BASE: baseline
GET: gas exchange threshold
GET60+: subjects with GET above 60 % $\overset{.}{\mathrm{V}}{\mathrm{O}}_{2\mathrm{peak}}$
GET60−: subjects with GET below 60 % $\overset{.}{\mathrm{V}}{\mathrm{O}}_{2\mathrm{peak}}$
HHb: deoxygenated hemoglobin
HIIT: high intensity interval training
HIT: high intensity training
NIRS: near-infrared spectroscopy
$$ \overset{.}{\mathrm{V}}{\mathrm{O}}_2 $$: oxygen uptake
O_2_Hb: oxygenated hemoglobin
${\overset{.}{\mathrm{V}}\mathrm{O}}_{2 \max }$: maximal oxygen uptake
$\overset{.}{\mathrm{V}}{\mathrm{O}}_{2\mathrm{peak}}$: peak oxygen uptake
OS: postexercise overshoot
THb: total hemoglobin
